# Route to Intelligent Imaging Reconstruction via Terahertz Nonlinear Ghost Imaging

**DOI:** 10.3390/mi11050521

**Published:** 2020-05-20

**Authors:** Juan S. Totero Gongora, Luana Olivieri, Luke Peters, Jacob Tunesi, Vittorio Cecconi, Antonio Cutrona, Robyn Tucker, Vivek Kumar, Alessia Pasquazi, Marco Peccianti

**Affiliations:** Emergent Photonics (EP*ic*) Laboratory, Department of Physics and Astronomy, University of Sussex, Brighton BN1 9QH, UK; jt420@sussex.ac.uk (J.S.T.G.); l.olivieri@sussex.ac.uk (L.O.); l.peters@sussex.ac.uk (L.P.); jt298@sussex.ac.uk (J.T.); v.cecconi@sussex.ac.uk (V.C.); ac878@sussex.ac.uk (A.C.); rt232@sussex.ac.uk (R.T.); vk230@sussex.ac.uk (V.K.); a.pasquazi@sussex.ac.uk (A.P.)

**Keywords:** terahertz, nonlinear optical conversion, complex optical systems, adaptive imaging, single-pixel imaging, surface nonlinear photonics

## Abstract

Terahertz (THz) imaging is a rapidly emerging field, thanks to many potential applications in diagnostics, manufacturing, medicine and material characterisation. However, the relatively coarse resolution stemming from the large wavelength limits the deployment of THz imaging in micro- and nano-technologies, keeping its potential benefits out-of-reach in many practical scenarios and devices. In this context, single-pixel techniques are a promising alternative to imaging arrays, in particular when targeting subwavelength resolutions. In this work, we discuss the key advantages and practical challenges in the implementation of time-resolved nonlinear ghost imaging (TIMING), an imaging technique combining nonlinear THz generation with time-resolved time-domain spectroscopy detection. We numerically demonstrate the high-resolution reconstruction of semi-transparent samples, and we show how the Walsh–Hadamard reconstruction scheme can be optimised to significantly reduce the reconstruction time. We also discuss how, in sharp contrast with traditional intensity-based ghost imaging, the field detection at the heart of TIMING enables high-fidelity image reconstruction via low numerical-aperture detection. Even more striking—and to the best of our knowledge, an issue never tackled before—the general concept of “resolution” of the imaging system as the “smallest feature discernible” appears to be not well suited to describing the fidelity limits of nonlinear ghost-imaging systems. Our results suggest that the drop in reconstruction accuracy stemming from non-ideal detection conditions is complex and not driven by the attenuation of high-frequency spatial components (i.e., blurring) as in standard imaging. On the technological side, we further show how achieving efficient optical-to-terahertz conversion in extremely short propagation lengths is crucial regarding imaging performance, and we propose low-bandgap semiconductors as a practical framework to obtain THz emission from quasi-2D structures, i.e., structure in which the interaction occurs on a deeply subwavelength scale. Our results establish a comprehensive theoretical and experimental framework for the development of a new generation of terahertz hyperspectral imaging devices.

## 1. Introduction

In recent years, there has been increasing interest in the development of imaging techniques that are capable of reconstructing the full-wave properties (amplitude and phase) of arbitrary electromagnetic field distributions [1,2,3]. While standard optical technologies, such as cameras and photodiodes, are usually sensitive to the field intensity, a large part of the sample information is encoded in the optical phase of the scattered field [4]. Interestingly, the direct detection of the field evolution is achievable at terahertz (THz) frequencies thanks to the availability of the time-domain spectroscopy (TDS) technique. TDS detection provides a time-resolved measurement of the electric field (e.g., via electro-optical sampling [5]), allowing researchers to retrieve the complex-valued dielectric function of a sample. Such a capability, coupled with the existence of specific and distinctive spectral fingerprints in the terahertz frequency range, are critical enabling tools for advanced applications, such as explosive detection, biological imaging, artwork conservation and medical diagnosis [6,7,8,9,10]. However, despite the vast body of potential applications, the development of TDS devices that are capable of high-resolution imaging is still regarded as an open challenge. A typical TDS implementation relies on complex and expensive optical components that cannot be easily integrated into high-density sensor arrays [11]. 

To date, THz imaging mostly relies on thermal cameras, essentially the equivalent of optical cameras, which employ arrays of micro-bolometers to measure the time-averaged intensity of the THz signal. As such, they cannot be employed for time-resolved THz detection and they are insensitive to the optical phase and temporal delay of the transmitted THz field. In an attempt to develop arrays of TDS detectors, researchers have proposed two-dimensional full-wave imaging devices that are composed of arrays of photoconductive antennas or Shack–Hartmann sensors [12,13]. However, these devices require complex and expensive technological platforms and their practicality is still a matter of debate. Furthermore, they fundamentally sample the image information in an array of single and well-separated small points. Hence, obtaining a high resolution can still require mechanical action on the sample.

A promising alternative to TDS imaging arrays is single-pixel imaging, or ghost imaging (GI). In these approaches, the sensor array is replaced by a single bucket detector, which collects the field scattered by the sample in response to a specific sequence of incident patterns. By correlating each acquired signal with its corresponding incident field distribution, it is possible to reconstruct the sample image [14,15,16,17]. However, despite its simplicity, the implementation of GI at terahertz frequencies is affected by the limited availability of wavefront-shaping devices (e.g., spatial light modulators) that are capable of impressing arbitrary patterns on an incident THz pulse. Following the initial experimental demonstrations with metallic masks and metamaterial devices [18,19], several research groups’ researchers have proposed indirect patterning techniques for the generation of high-resolution THz patterns. One of the most successful approaches relies on the generation of transient photocarrier masks on semiconductor substrates [20,21,22,23]. In these experiments, a standard optical Spatial Light Modulator (SLM) impresses a spatial pattern on an ultrafast optical beam. Upon impinging on a semiconductor substrate, the latter generates a distribution of carriers matching the desired pattern profile, which acts as a transient metallic mask and can be used to pattern an external THz beam. While this technique has been successfully employed to achieve THz imaging with a deeply subwavelength resolution, it is also affected by a few limitations. In particular, recent works have shown that the maximum resolution achievable with these techniques is strongly dependent on the semiconductor substrate thickness: in Stantchev and coworkers [20,21], for example, researchers have demonstrated that deeply subwavelength resolutions are achievable only when considering patterning substrates with a thickness below 10 µm.

In a series of recent works, we have proposed a new imaging technique, time-resolved nonlinear ghost imaging (TIMING), which overcomes several of these limitations [24,25,26]. TIMING relies on the integration of nonlinear THz pattern generation with TDS single-pixel field detection. In this work, we discuss the main features of our approach and present our latest results on the theoretical framework underlying our image reconstruction process. Via analysis of the compression properties of the incident pattern distribution, we show how a TIMING implementation based on an optimised Walsh–Hadamard encoding scheme can significantly reduce the number of incident patterns required to obtain a high-fidelity image of the sample. Finally, we discuss how the development of ultra-thin THz emitters can provide a significant improvement to the imaging performance of TIMING.

## 2. Time-Resolved Nonlinear Ghost Imaging: A Conceptual Overview

**Figure 1:**
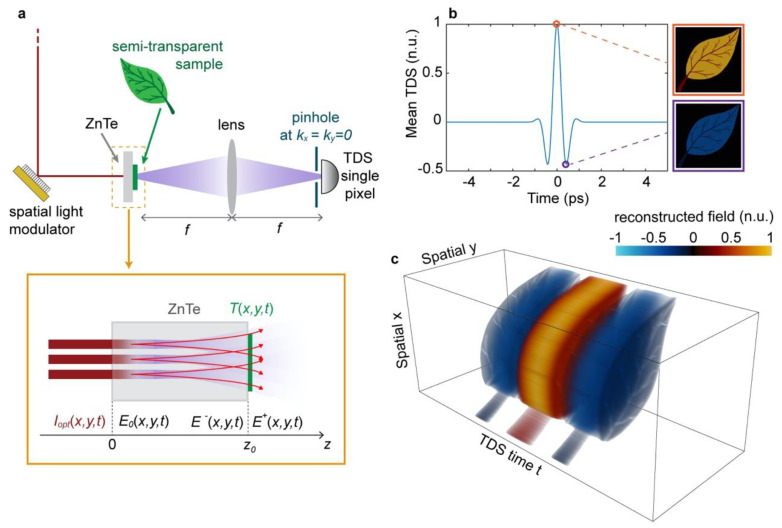
Conceptual description of time-resolved nonlinear ghost imaging (TIMING). (**a**) Schematic of the experimental setup. (**b**,**c**) Simulation of the TIMING reconstruction of a semi-transparent sample, including the average field transmission (panel b) and the full spatiotemporal image of the sample (panel c). The simulated object size was 10.24 cm × 10.24 cm, sampled with a spatial resolution of 512 × 512 pixels (Δ*x =* 200 µm) and a temporal resolution of Δ*t* = 19.5 fs. The nonlinear crystal thickness was $z_{0}\;$= 10 μm. n.u.: normalised units, TDS: Time-domain spectroscopy.

A conceptual schematic of our imaging setup is shown in Figure 1a. A spatial pattern is impressed on the optical beam through a binary spatial light simulator, e.g., a digital micromirror device (DMD), obtaining the optical intensity distribution $I_{n}^{opt}\left(x,y,\omega \right)$. The THz patterns $E_{n}^{0}\left(x,y,t\right)$ are generated using a nonlinear conversion of $I_{n}^{opt}\left(x,y,\omega \right)$ in a nonlinear quadratic crystal (ZnTe) of thickness $z_{0}$. The THz pattern propagates across the crystal and interacts with the object, yielding a transmitted field, which is collected by a TDS detection setup. Different from the standard formulations in optics, which relies on the optical intensity, our object reconstruction scheme relies on the time-resolved detection of the electric field scattered by the object. More specifically, the electric field distribution is defined immediately before and after the object as $E^{-}(x,y,t=E\left(x,y,z_{0}-\epsilon ,t\right)$ and $E^{+}\left(x,y,t\right)=E\left(x,y,z_{0}+\epsilon ,t\right)$, respectively, where $z_{0}$ is the object plane and ϵ>0 is an arbitrarily small distance (Figure 1a, inset). Without loss of generality, the transmission properties of the object are represented by defining the transmission function T(x,y,t), which is defined on both the spatial and temporal components to account for the spectral response of the sample. To simplify our analysis, in the following, we considered two-dimensional objects, i.e., we restricted ourselves to transmission functions of the form T(x,y,t). Under this position, the transmitted field is straightforwardly defined as:

(1) $$E^{+}\left(x,y,t\right)=\int dt^{\prime }T\left(x,y,t-t^{\prime }\right)E^{-}\left(x,y,t\right).$$

The objective of a single-pixel imaging methodology is to reconstruct the transmitted field distribution $E^{+}\left(x,y,t\right)$ through a sequence of measurements to retrieve the transmission function of the object. In our approach, this corresponds to measuring the TDS trace of the spatially-averaged transmitted field from the object in response to a sequence of predefined patterns (a procedure known as computational ghost imaging) [27]. The nth pattern is denoted by $E_{n}^{-}\left(x,y,t\right)=P_{n}\left(x,y\right)f\left(t\right)$, where $P_{n}\left(x,y\right)$ is the deterministic spatial distribution of the pattern and f(t) is the temporal profile of the THz pulse. The reconstruction process is defined as follows:

(2) $$T\left(x,y,t\right)=C_{n}\left(t\right)P_{n}^{}\;{\left(x,y\right)}_{n}-C_{n}{\left(t\right)}_{n}P_{n}{\left(x,y\right)}_{n}\;,$$

where ${\langle \cdots \rangle }_{n}$ represents an average over the distribution patterns and the expansion coefficients $C_{n}\left(t\right)$ are defined as follows:

(3) $$C_{n}\left(t\right)=\int dxdy\;E_{n}^{+}\left(x,y,t\right)=\int dxdydt^{\prime }T\left(x,y,t-t^{\prime }\right)E_{n}^{-}\left(x,y,t\right).$$

A numerical implementation of the image reconstruction process is shown in Figure 1b,c, where we employed TIMING to reconstruct the transmitted field from a semi-transparent sample (a leaf). In Figure 1b, we report the spatial average of the reconstructed field, exhibiting the characteristic temporal profile of the incident THz pulse. Since our image reconstruction operates simultaneously in time and space, it allows for not only retrieving the spatial distribution of the object but also its temporal/spectral features. The specific result of a TIMING scan is a spatiotemporal image of the transmitted field, as shown in Figure 1c.

An interesting question is whether the distance between the distribution of THz sources and the sample has any effect on the image reconstruction capability of our setup. This point is pivotal when time-resolved imaging is desired, as propagation always induces space–time coupling. This condition represents a typical challenge in mask-based ghost imaging when time-domain detection is sought. The propagation within the patterning crystal is known to lead to significant reconstruction issues when considering deeply subwavelength patterns [20,21,22]. These issues are related to the intrinsic space–time coupling that takes place within the crystal [28]. In essence, once the patterns are impressed on the THz wave at the surface of the crystal (at z=0), they undergo diffraction. As a result, the electric field distribution $E_{n}^{-}\left(x,y,t\right)$ probing the sample is not the initial distribution $E_{n}^{0}\left(x,y,t\right)$, but rather a space-time propagated version of it. The latter is mathematically expressed as:

(4) $$\;E_{n}^{-}\left(x,y,t\right)=E_{n}^{}\left(x,y,z_{0}-\epsilon ,t\right)=\int dxdydt^{\prime }G\left(x-x^{\prime },y-y^{\prime },z_{0}-\epsilon ,t-t^{\prime }\right)E_{n}^{0}\left(x,y,t\right),$$

where $G\left(x,y,z_{0}-\epsilon ,t\right)$ is the dyadic Green’s function propagating the field from z=0 to $z=z_{0}-\epsilon$. Since space–time coupling is essentially a linear process, it can be inverted by applying a Weiner filter to the reconstructed image to mitigate the effects of diffraction. In the angular spectrum coordinates $\left(k_{x},k_{y},z,\omega \right)$, the Weiner filter is defined as:

(5) $$W\left(k_{x},k_{y},z,\omega \right)=\frac{G^{†\;}\left(k_{x},k_{y},z,\omega \right)}{\left|G\left(k_{x},k_{y},z,\omega \right)\right|+\alpha NSR\left(k_{x},k_{y},\omega \right)},$$

where $NSR\left(k_{x},k_{y},\omega \right)$ is the spectral noise-to-signal distribution, α is a noise-filtering fitting parameter and † stands for Hermitian conjugation [24]. As expressed by Equation (5), the Weiner filter is the equivalent of an inverse Green’s function operator that is modified to take into account the presence of noise in the experimental measurements. The effect of the NSR term in the denominator, which is controlled by the parameter α, is to suppress the regions of the spectrum that are dominated by noise and could render the inversion operation an intrinsically ill-posed problem [29]. 

From a physical point of view, Equations (4) and (5) can be read as follows: when performing a time-domain reconstruction of the image, the spatial distribution of $\;E_{n}^{+}\left(x,y,t\right)$ is acquired at a given time. However, this is not the scattered field from the object in response to the incident pattern $E_{n}^{0}$ at that time; there is no time in which the scattered field $E_{n}^{0}\left(x,y,t\right)$ is univocally represented in the sampling pattern$\;E_{n}^{-}$. The reason is simply that the method is slicing a fixed-time contribution of a piece of information that is warped in the space-time. This warping is introduced by the distance between sources and the object plane; therefore, it is different for any plane of the object being imaged.

Said differently, using fixed-time images to reconstruct planar features produces a fundamentally incorrect picture of the evolving scattered field, with different degrees of “distortion” introduced by the amount of propagation. It is worth noting that, although related, this is not the same concept as that of resolution degradation of incoherent near-field systems. In fact, Equation (4) shows that any space-time information retained by the field can be accessed only by accounting for near-field propagation. TIMING reconstructs the image of a scattered field from an object with fidelity by applying the backpropagation kernel from Equation (5). Another interesting aspect is whether the thickness of the nonlinear crystal accounts for an overall separation between terahertz sources and the object, affecting the achievable resolution. The difference here is that the propagation is inherently nonlinear and although the generated terahertz signal diffracts linearly, for any desired resolution, there is always a given generating crystal section that is sufficiently close to the object to illuminate it within the required near-field condition. We have recently theoretically and experimentally demonstrated that the diffraction limit does not directly apply in the nonlinear GI via the generation crystal thickness since the nonlinear conversion from optical to THz patterns is a process distributed across the crystal [25]. We argue that this general approach is particularly useful when considering samples stored in cuvettes or sample holders.

## 3. Compressed and Adaptive Sensing Applications

In this section, we discuss the image reconstruction performance of TIMING as a result of our particular choice of input pattern distribution. To reconstruct the sample, TIMING relies on the Walsh–Hadamard (WH) image decomposition, which constitutes the binary counterpart of standard Fourier-based image analysis [30]. In our approach, the choice of the incident pattern distribution was driven by three considerations: (i) the compatibility with the available wavefront-shaping technology impressing patterns on the optical beam, (ii) the average signal-to-noise ratio (SNR) of the signal associated with each incident pattern and (iii) the energy compaction (compressibility) properties of the image expansion base. The WH patterns can be implemented straightforwardly through a digital micromirror device (DMD) and they are known to maximise the SNR of the acquired signals in experiments [31,32]. The latter is a significant advantage when compared to standard TDS imagers, which rely on a raster-scan reconstruction approach, where either the source or receiver (or both) are sequentially moved across the sample, leading to a combination of single-pixel detection and illumination [10]. While this approach is intuitive and straightforward to implement, a single-pixel illumination usually implies a degradation of the SNR of the expansion coefficients for a fixed intensity per pixel. Furthermore, raster-scan imaging is a local reconstruction algorithm that is not suitable for compressed sensing; in mathematical terms, the raster scan corresponds to expanding the sample image in the canonical Cartesian base $E_{n,m}\left(x,y\right)=\delta \left(x-x_{n},y-y_{n}\right)$. Trivially speaking, to reconstruct the entire image with this approach, each pixel composing it needs to be scanned. 

In contrast, the WH encoding scheme is a very popular example of energy compacting (compressive) decomposition, as in the case of Fourier-based or wavelet-based image analysis [33,34]. In these approaches, the image is represented as an orthogonal basis of extended spatial functions. For example, in the case of Fourier image analysis, the sampling patterns are the basis of the two-dimensional Fourier Transform [29,35]. The choice of an expansion basis composed of extended patterns has two main advantages. First, extended patterns are generally characterised by transmitted fields with higher SNRs because distributed sources generally carry more power. In fact, for a given power limit per pixel, the Walsh–Hadamard decomposition allows for a total energy per pattern that is about N/2 higher than single-pixel illumination. Second, and more importantly, there is no one-to-one correspondence between individual image pixels and distinct measurements (as in the case of the raster scan). In fact, the incident patterns not only probe different parts of the sample in parallel but can also provide useful insights into its spatial structure, even before completing the entire set of illuminating patterns.

**Figure 2:**
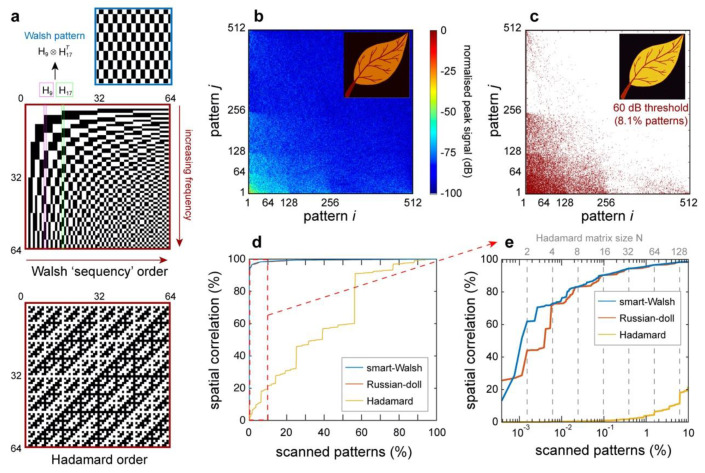
Walsh–Hadamard image reconstruction. (**a**) Generation of incident patterns from the Walsh–Hadamard matrix. Each pattern is defined as the tensor product between two columns of the generating matrix. The patterns can be generated from different configurations of a Hadamard matrix: we show the Walsh, or “sequency”, order (top, used in TIMING) and the standard Hadamard, or “natural”, order (bottom). (**b**,**c**) Reconstructed Walsh spectrum of the peak-field object transmission. Interestingly, only a fraction of the patterns (8.1%) were associated with a spectral amplitude exceeding the −60 dB threshold (with 0 dB being the energy correlation of the fittest pattern—panel c). Nevertheless, these patterns were sufficient to provide a high-fidelity reconstruction of the image (insets). (**d**,**e**) Pearson correlation coefficients between reconstructed and original images as a function of the number of patterns employed in the reconstruction. The results refer to the entire scan (panel d) and the initial 10% of patterns (panel e).

In practical terms, a WH pattern of size N×N is obtained by considering the tensor product between the columns (or, invariantly, rows) of the corresponding N×N Walsh–Hadamard matrix (see Figure 2a). The columns (or rows) are mutually orthogonal and form a complete tensor basis for any two-dimensional matrix. Interestingly, the columns of the Hadamard matrix can be re-arranged in different configurations, leading to matrices with different orderings [36,37,38]. In Figure 2, we compare two configurations: the Walsh (or sequency) order and the Hadamard (or natural) order. The Walsh ordering is particularly useful in image reconstruction as it mirrors the standard order of the discrete Fourier basis, i.e., the columns are sorted in terms of increasing spatial frequencies. This means that by using the Walsh matrix, it is possible to acquire complete lower-resolution images before completing the illumination set, which can be useful for applying decisional approaches and reducing the set dimension [39,40]. 

To illustrate how the image information is distributed across the basis of incident patterns, it is useful to analyse the peak-field Walsh spectrum of the reconstructed image, which is shown in Figure 2b. The WH spectrum is obtained by plotting the $C_{n}\left(t=t_{peak}\right)$ coefficients as a function of their generating pattern indexes. As can be evinced from Figure 2b, the WH decomposition re-organises the image information into a hierarchical structure, which mirrors the spectral content of the image. Interestingly, this property is at the core of the compression properties of the WH encoding scheme, as can be exploited to significantly reduce the number of measurements required to reconstruct the image. We illustrate this result in Figure 2c, where we identify the coefficients with an amplitude exceeding a −60 dB threshold with a red marker. As shown in Figure 2c, these significant coefficients were mostly localised in correspondence with the smaller spatial frequencies of the image, and for this image, they represented 8.1% of the total number of patterns. Remarkably, this limited number of patterns was sufficient to accurately reconstruct the image (as shown in Figure 2c, inset). 

For a given Walsh–Hadamard matrix, it is also critical to consider the specific order employed when selecting the sequence of columns forming the distribution of incident patterns. In our approach, we implemented an optimised ordering of the WH patterns (denoted as “smart-Walsh”), which sorts the incident patterns in terms of increasing spatial frequency (see Supplementary Video 1). In Figure 2d,e, we illustrate the fidelity of the TIMING reconstruction across the ensemble of incident patterns for different sorting schemes. The fidelity between reconstructed and original images is estimated through the Pearson correlation coefficient, which measures the spatial correlation between the two datasets and is defined as:

(6) $$\rho \left(A,B\right)=\frac{\sum_{mn}^{}\left(A_{mn}-\bar{A}\right)\left(B_{mn}-\bar{B}\right)}{\sqrt{\sum_{mn}^{}{\left(A_{mn}-\bar{A}\right)}^{2}\cdot \sum_{mn}^{}{\left(B_{mn}-\bar{B}\right)}^{2}}},$$

where $\bar{A}$ and $\bar{B}$ are the spatial averages of A and B, respectively. In our analysis, we considered the performance of our “smart-Walsh” sorting (blue line) with the natural Hadamard sorting (yellow line) and the recently proposed “Russian-doll” sorting (orange line) [38]. As shown in Figure 2d, both the smart-Walsh and the Russian-doll sorting were capable of high-fidelity reconstructions of the sample image, even just by using a fraction of patterns, especially when compared to the standard Hadamard case. Further insights on the image reconstruction performance can be obtained by analysing the image reconstruction across the first 10% of patterns (Figure 2e). Remarkably, both our approach and the Russian-doll sorting outperformed the standard Hadamard sorting, yielding a high-fidelity image (spatial correlation exceeding 90%) by considering only 0.1% of the total number of patterns. Interestingly, while the performance of our “smart-Walsh” approach matched the Russian-doll sorting as soon as each Hadamard order was completed (dashed grey lines), we observed that it outperformed it across incomplete scans. 

## 4. Performance of Field-Based Ghost-Imaging Detection in the Fourier Plane

The possibility of performing field-sensitive detection provides TIMING with a significant advantage when compared with traditional GI. However, the typical GI correlation between detection parameters and image fidelity is broken by the nonlinear ghost imaging transformation, i.e., the need for establishing a correlation between coherent-field detection and the optical intensity patterns. More precisely, the implementation of a field average in the image extraction radically changes the way the image quality depends on the experimental parameters. Standard GI reconstruction relies on detecting the integrated scattered field to estimate the spatial correlation between the incident patterns and the sample, where:

(7) $$C_{n}=\int dxdy\;I_{n}^{+}\left(x,y\right)=\int dxdydt^{\prime }{\left|T\left(x,y,t-t^{\prime }\right)E_{n}^{-}\left(x,y,t\right)\right|}^{2}.$$

This corresponds to the direct acquisition of the total scattered field with a standard bucket detector, which integrates the transmitted intensity distribution. Fundamentally, it is an estimator of the total scattered power, and as such, it is directly affected by the numerical aperture of the detector and by the distance between the detector and the sample. As discussed in the literature on optical GI, both these factors directly fix the amount of information that is available when reconstructing the image and directly affect its fidelity [15].

TIMING inherits the direct detection of the scattered THz field distribution from time-domain spectroscopy systems. By operating directly on the electric field, it allows for measuring the average THz scattered field (in a fully coherent sense) by performing a point-like detection in the Fourier plane. As defined by Equation (3), the coefficients $C_{n}$ can be obtained by measuring the $\left(k_{x},k_{y}\right)=0$ spectral components of the THz transmitted field:

(8) $$C_{n}\left(t\right)=\int dxdy\;E_{n}^{+}\left(x,y,t\right)=ℱ\left[E_{n}^{+}\left(x,y,t\right)\right]\;{\;|}_{k_{x}=0,\;k_{y}=0}.$$

This implementation implies that the experimental measurement of the correlations $C_{n}$ is not limited at all by the numerical aperture of the bucket detector. This type of measurement can be obtained by placing the object in the focal point of an arbitrary lens and by acquiring the signal in the central point of the opposite focal plane (Figure 1a). The electric field in the focal plane reads as follows:

(9) $$E_{focal}\left(x{,}^{\prime }y^{\prime }\right)\propto \;ℱ\left[E_{n}^{+}\left(x,y,t\right)\right]\;\left(k_{x}=\frac{x^{\prime }}{\lambda f},k_{y}=\frac{y^{\prime }}{\lambda f}\right),$$

where $x^{\prime }$ and $y^{\prime }$ are the physical coordinates in the Fourier plane [41].

However, in terms of implementation, the detector samples a finite small area of the Fourier plane with an area-sampling function $PH\left(k_{x},k_{y}\right)$, obtaining the estimation $C_{n}{}^{\prime }\left(t\right)$:

(10) $$C_{n}^{{}^{\prime }\left(t\right)}=\underset{}{\int }PH\left(k_{x},k_{y}\right)*\;ℱ\left[E_{n}^{+}\left(x,y,t\right)\right]\;dk_{x}dk_{y},$$

where $PH\left(k_{x},k_{y}\right)$ is physically represented by the profile of the probe beam in the electro-optical sampling (e.g., a Gaussian function), or by the shape of any aperture implemented in front of the nonlinear detection to fix its interaction area with the THz field. 

The accuracy of the measurements is then directly related to how “point-like” our detection can be made. Although one could be tempted to foresee a general benefit of the high signal-to-noise ratio (SNR) resulting from large detection apertures as in the standard GI, this is also a source of artefacts, fundamentally establishing a trade-off between SNR and fidelity.

**Figure 3:**
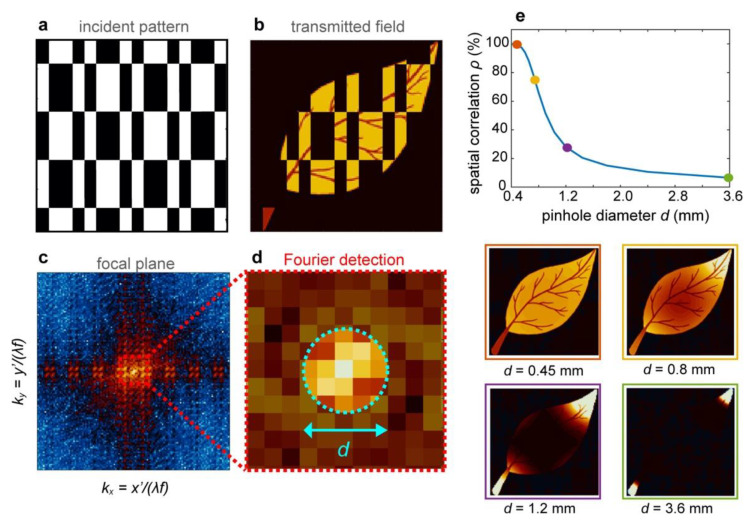
Influence of the pinhole size on the Fourier detection of TIMING reconstruction coefficients. **(a**–**d**) The spatial average of the transmitted field (**b**) associated with each incident pattern (**a**) could be measured by performing a point-like detection in the centre of the Fourier plane (**c**,**d**). In realistic implementations, the centre of the Fourier plane is sampled using a sampling function *PH* of finite diameter *d*. (**e**) Spatial correlation between the reconstructed and original image as a function of the sampling function diameter. A departure from the point-like approximation led to a significant corruption of the reconstructed image (insets). Interestingly, the typical image degradation did not necessarily involve the total disappearance of highly resolved details.

Figure 3 illustrates the effects of the size *d* of the sampling function $PH\left(x^{\prime }=k_{x}\lambda f,y{}^{\prime }=k_{y}\lambda f\right)$ on the image reconstruction fidelity (Figure 3e). Interestingly, the reduction of fidelity observed for increasing the sampling diameter is different from the typical limitations in standard imaging. In our case, a too-large area sampling function in the Fourier plane did not lead to a reduction in the discernible details but rather in the disappearance of entire parts of the image (see Figure 3e, insets). 

**Figure 4:**
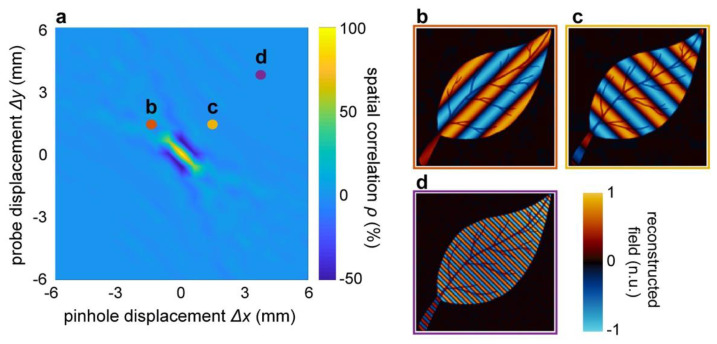
Influence of the pinhole displacement on the Fourier detection of TIMING reconstruction coefficients. (**a**) Spatial correlation between the reconstructed and original image as a function of the sampling function position in the focal plane. The displacement *(Δx, Δy)* was measured with respect to the lens axis and the sampling function diameter was set to *d* = 0.36 mm, corresponding to a spatial correlation of 100% at the centre of the Fourier plane (cf. Figure 3e). (**b**–**d**) Examples of image reconstruction with off-axis detection, illustrating the appearance of spurious spatial frequencies. Interestingly, the object morphology was still noticeable, even at a relatively large distance from the optical axis.

Similarly, in Figure 4, we illustrate the effect of a misalignment of the sampling function *PH* centre with respect to the centre of the Fourier plane. Trivially, the spatial correlation between the reconstructed and original images peaks at the centre of the Fourier plane and swiftly decayed in the case of off-axis detection (Figure 4a). In these conditions, the reconstructed image showed the appearance of spurious spatial frequencies, corresponding to the $\left(k_{x},\;k_{y}\right)$ sampling position (Figure 4b,d). Interestingly, however, the overall morphology and details of the image were still present in the images, and no noticeable blurring occurred.

## 5. A Route towards Thinner THz Emitters: Surface Emission from Quasi-2D Semiconductor Structures

Deep near-field regimes are in general a requirement to obtain deep-subwavelength image resolutions. Here, we review this current technological solution that is under development in TIMING towards this goal.

In terms of nonlinear ghost imaging, the high resolution fundamentally results from the ability to achieve significant optical-to-terahertz conversions, keeping the sample in the proximity of the distribution of terahertz sources. This translates into the need for generating terahertz from quite thin devices (although we argued how TIMING exhibits significantly more relaxed constraints compared to previous literature [25]).

Although the technology is continuously evolving, the best-performing and most practical off-the-shelf sources are within the class of electro-optical switches. The terahertz emission is generated by a transient current that is sustained by an external electric source and is triggered by a change of conductivity induced by an ultrafast optical absorption [5]. This specific approach benefits from a virtually high optical-to-terahertz conversion efficiency since the actual source of radiation is a current sustained by the electric source. However, this technology is difficult to translate to TIMING since the integration into a single device of a dense distribution of independent electrical switches emitting terahertz signals is extremely challenging. 

In terms of direct optical-to-terahertz conversion, improving the efficiency of nonlinear converters is undoubtedly a central research area with a vast spectrum of proposed solutions ranging from novel materials to the design of sophisticated propagation geometries, which allows for very long interaction lengths. However, very few alternatives are currently available for emitters with a thickness below the micrometre scale. One general issue is that the efficiency of bulk nonlinear interactions tend to be vanishingly low at this scale, whereas the ruling mechanisms of the nonlinear interactions are dominated by peculiar physical mechanisms that exist only in quasi-2D frameworks. Some very promising, recently explored solutions comprise exploiting spin-mediated current transients (spintronic emitters) in nano-hetero-metallic structures [42]. On the other hand, a significant fraction of the work in this research area focuses on achieving a very large interfacial nonlinear response or inducing carrier-mediated nonlinear dynamics at a surface. 

**Figure 5:**
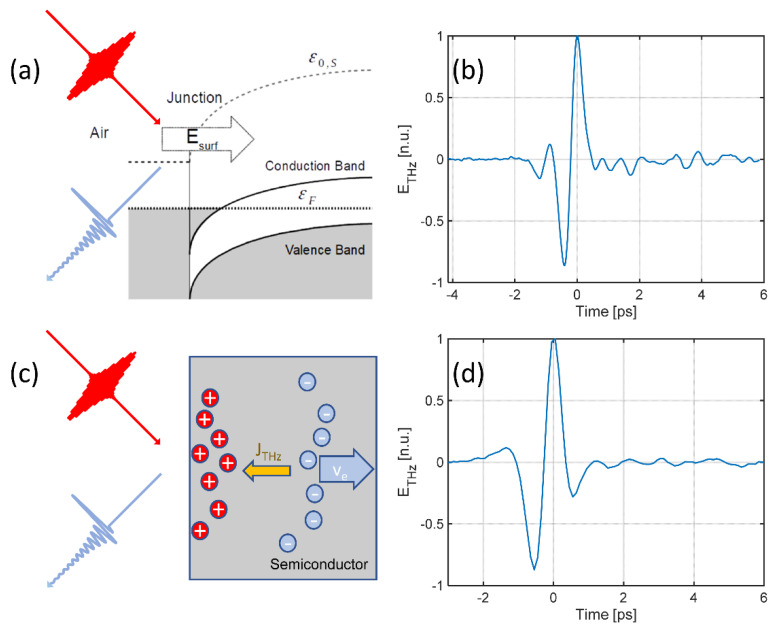
Surface emission driving mechanisms. (**a**) Surface optical rectification—a surface field at the air–semiconductor barrier combines with the optical field in a four-wave mixing process (cubic), generating a terahertz mixing product (see Equation (7)). (**b**) Measurement of the terahertz emission using surface optical rectification with an optical pulsed excitation fluence of 7 mJ/cm^2^ (1 kHz repetition rate) and a pulse with a wavelength of 800 nm and a duration of 90 fs. (**c**) Simplified sketch of the photo-Dember process in InAs. The absorption of an ultrashort pulse generates a high density of photogenerated hole–electron pairs within the optical penetration depth (140 nm). The fast diffusion of the electrons induces a transient current *J*_THz_, which is the source of the terahertz emission. (**d**) Measurement of the terahertz emission by photo-Dember mechanism with an optical pulsed excitation fluence of 0.28 µJ/cm^2^ (80 MHz repetition rate) and pulse with a wavelength of 800 nm and a duration of 140 fs.

In general, these effects are fundamentally driven by breaking the lattice symmetry, which is produced by the material discontinuity at the interface. The requirement of tightly reduced interaction lengths makes low-bandgap semiconductors, such as Indium Arsenide (InAs) and Indium Antimonide (InSb), very popular experimental frameworks. What motivated the interest in these systems is the surprisingly high conversion efficiency per interaction length [43,44,45]. In a traditional NIR ultrafast excitation setting, the mean absorption length for photons is very small, typically within the scale of $l_{d}$ = 140 nm at a wavelength λ = 800 nm. At low fluences (below 100 nJ/cm^2^), InAs is probably considered the benchmark surface emitter. In this case, the generation is driven by the very large difference in mobility between holes and electrons via the photo-Dember effect (Figure 5c,d): when a high density of photogenerated pairs is induced in the proximity of the surface, electrons quickly diffuse away from the surface, leaving uncompensated carriers of the opposite sign. Such a charge unbalance creates a fast stretching dipole, or equivalently, a local current transient that is the source of the terahertz emission [46]. 

At very high pumping energies (above 10 μJ/cm^2^), this phenomenon becomes critically saturated due to the electromagnetic screening role of dense carrier densities. Conversely, the optical surface rectification (SOR) dominates the emission [43]. The optical surface rectification is a quadratic phenomenon induced by the contribution of a local static field at the surface, which is induced by surface states within the bulk cubic nonlinear response (Figure 5a,b). The DC field effectively plays the role of a field contribution in a four-wave mixing process in a mechanism commonly referred to as a field-induced quadratic response [45,47] and is described using:

(11) $$E_{THz}\propto \chi^{\left(3\right)}E_{surf}E_{\omega }^{*}E_{\omega },$$

where $\chi^{\left(3\right)}$ is the third-order susceptibility of InAs, $E_{surf}$ is the intrinsic surface potential field, $E_{\omega }$ is the incident optical field and ∗ stands for the complex conjugate. Quite interestingly, because the phenomenon is driven by a surface potential, it is also a measurable way to probe the dynamics of the carrier at the surface, and it has been proposed as the optical analogy of a Kelvin probe [48].

## 6. Discussions and Conclusions

In this work, we have provided an overview of the advantages and implementation challenges of a time-resolved nonlinear ghost-imaging approach to THz single-pixel imaging. By combining nonlinear THz generation and single-pixel TDS detection, we demonstrated the high-resolution reconstruction of a semi-transparent sample with a subwavelength resolution (512 × 512 pixels). By providing a detailed analysis of the Walsh–Hadamard reconstruction scheme, we have shown how a specific choice of patterns and the order of acquisition can play a beneficial role in speeding-up the reconstruction of the peak-field transmission from the sample. Remarkably, we have shown that less than 10% of the incident samples were required to achieve a high-fidelity reconstruction of the sample image in a general sequential reconstruction. Our approach, which is based on a lexicographical sorting of the incident patterns in terms of their spatial frequency (an approach we denoted as a “smart-Walsh” reconstruction), is general and image-independent and can be applied to reduce the overall reconstruction time for unknown samples. Interestingly, such a result could be further improved by considering that even a smaller percentage of incident patterns are required to reconstruct the sample: in our case, only 8% of the patterns were associated with an expansion coefficient exceeding 60dB. In practical terms, this would correspond to a 92% shorter acquisition time, corresponding to a 12.5× speed up of the image reconstruction process when compared to a full scan based on the Hadamard encoding scheme. These numbers suggest that the reconstruction process could be significantly sped up through the application of adaptive-basis-scan algorithms and deep-learning-enhanced imaging, which identify and predict the best set of scanning patterns in real time [40,49,50,51]. 

Interestingly, our results suggest that the nonlinear GI methodology is not limited by the numerical aperture of the optical system in a “conventional” sense. Said differently, it operates under the assumption of a very low numerical aperture to obtain a faithful spectral representation of the image. However, our results highlight that the image reconstruction is quite sensitive to the size and alignment of the pinhole function selecting the $\left(k_{x},k_{y}\right)=0$ components of the scattered field. Most importantly, in sharp contrast with previous literature on the topic, the reconstruction accuracy cannot simply be represented as a matter of effective “resolution”. The drop in reconstruction fidelity, in fact, is not driven by the attenuation of high-frequency spatial components (i.e., blurring) as in standard imaging, but it can lead to the appearance of artefacts and spurious spatial frequencies. To the best of our knowledge, the reconstruction limits of single-pixel time-domain imaging have never been formalised elsewhere. 

Finally, although thin emitters are a general requirement for this approach, TIMING exhibits relaxed constraints between the nonlinear interaction length and the image resolution. Yet, solutions for sub-micron-thick large-area terahertz generation are practically possible, enabling resolutions within the same scale or better. A promising platform to achieve this goal is narrow-bandgap semiconductor devices based on InAs or InSb platforms. These materials not only provide extremely high optical-to-terahertz conversion efficiency per unit length but they are also suitable for large-scale fabrication and deployment in real-world devices thanks to their established deployment in the electronic domain.

We believe that TIMING is a significant step forward in the development of terahertz micro-diagnostics based on hyperspectral imaging devices. Our approach also addresses fundamental criticalities in the imaging reconstruction process, which generally affect any high-resolution imaging domain where high temporal resolution is sought. As such, TIMING establishes a comprehensive theoretical and technological platform that paves the way for new generations of terahertz imaging devices satisfying the requirements for high-resolution and spectral sensitivity in real-world applications. 

## Supplementary Materials

The following are available online at https://www.mdpi.com/2072-666X/11/5/521/s1.
